# Profiles of Risky Sexual Behaviors and Associated Factors Among Sexually Active Men in Rwanda: A Nationwide Survey

**DOI:** 10.1007/s10508-026-03458-6

**Published:** 2026-05-29

**Authors:** Joseph Kawuki, Quraish Sserwanja, Jean H. Kim

**Affiliations:** 1 Jockey Club School of Public Health and Primary Care, Faculty of Medicine, The Chinese University of Hong Kong, Hong Kong, SAR, China; 2 Program in Public Health, Department of Family, Population, and Preventive Medicine, Stony Brook University, Stony Brook, NY USA; 3 Programmes Department, Relief International, Khartoum, Sudan

**Keywords:** Risky sexual behaviors, Men, Sexually active, HIV risk, Rwanda Demographic and Health Survey

## Abstract

Risky sexual behaviors (RSB) remain one of the major causes of unintended pregnancies and sexually transmitted infections. This study aimed to assess the prevalence of various RSB and their correlates among sexually active men in Rwanda, one of the poorest countries in the world. Using data from the 2020 Rwanda Demographic and Health Survey, a nationally representative sample of sexually active males (n = 4735) was examined for the prevalence of non-condom use, having multiple sex partners, engagement in commercial sex, and sexual debut before age 18. Multivariable logistic regression was conducted to explore 17 factors associated with various patterns of RSB. Results indicated that non-condom use, early sexual debut, multiple sex partners, and engagement in commercial sex were reported by 55.3%, 17.4%, 8.3%, and 7.3%, respectively. Older, married, and uncircumcised males with no internet access, and those from large female-headed households and the Western region, were more likely to engage in RSB without condoms (*AOR*: 1.28–6.20, *p* < 0.05). In contrast, those engaging in RSB with condoms were less likely to be married and from the Southern region (*AOR* = 0.03 and 0.52, both *p* < 0.05), but more likely to have a secondary education (*AOR* = 2.29, *p* < 0.05). Those reporting an early sexual debut were more likely to be younger, unmarried, from large households, with no education, and non-Catholic (*AOR*: 1.24–2.72, *p* < 0.05). There are differing groupings of Rwandan males that engage in various patterns of RSB that may partly reflect issues of access, changing cultural values, and geographic variations. These results indicate the need for segmented risk reduction interventions targeting each of these high-risk groups.

**Supplementary Information:**

The online version contains supplementary material available at 10.1007/s10508-026-03458-6.

## Introduction

Globally, over 40.8 million adults are currently living with HIV/AIDS, and more than two-thirds of these individuals live in sub-Saharan Africa (World Health Organization, 2024). The high prevalence of HIV and other sexually transmitted infections (STIs) in this region is largely attributable to risky sexual behaviors (RSB), which include behaviors such as having multiple sexual partners, inconsistent use/non-use of condoms, engaging in transactional sex, early sexual debut, and having sexual intercourse while under the influence of alcohol or other intoxicants (Darteh et al., 2020; Dixon-Mueller, 2008; Gebeyehu & Mulatie, 2021; Goicolea et al., 2010; Hervish & Clifton, 2012; Sserwanja et al., 2023). RSB is associated with not only a higher risk of STIs but also a greatly increased risk of unwanted pregnancies (Ali & Tadele, 2021; Gebeyehu & Mulatie, 2021; Sserwanja et al., 2023). RSB continue to be a major public health issue in sub-Saharan Africa, where over 20% of sexually active males and 10% of females are noted to have multiple sex partners, and early sexual debut is widespread (Darteh et al., 2020; Dixon-Mueller, 2008). Although condom use has increased in this region, the levels are not yet sufficient to counter the high levels of RSB (Goicolea et al., 2010; Hervish & Clifton, 2012).

With a per capita income of approximately USD 1000, Rwanda is one of the poorest countries in the world, where over 46% of the population is 18 years of age and below (mean age = 19.7 years) (World Bank Group, 2024; World Population Review, 2022). Recent data indicate that Rwanda's overall HIV prevalence among adults is 3% (Nsanzimana et al., 2022). Although several studies have looked at human sexual behaviors in both Rwanda and sub-Saharan Africa, the target populations of the majority of these studies have been adolescent females, youths, high-risk populations, and women, with little emphasis on men (Michielsen et al., 2014; Mutagoma et al., 2018, 2017; Mutanda & Odimegwu, 2017; Ntaganira et al., 2012; Workneh et al., 2024). A recent multi-country study of East African countries noted that over one-quarter of reproductive-aged women had engaged in RSB (Workneh et al., 2024), indicating that interventions are urgently needed to address this ongoing public health issue. As the majority of household heads in Rwanda are males (National Institute of Statistics of Rwanda [NISR] et al., 2021), and a large proportion of unions between couples are informal unions in which the females have few recognized legal rights, sexual decision-making is likely to rest heavily on the preferences of the males (Stern & Mirembe, 2017). Males in Rwanda are, therefore, important stakeholders in the prevention of unintended pregnancies, as their involvement directly impacts family planning decisions and contraceptive use. Furthermore, male involvement has been noted to be important for the success of reproductive health programs (Manirafasha & Nishimwe, 2024; United Nations Fund for Population Activities, 2022). Recent studies of Rwandan men have noted an STI prevalence of 6.21% among all reproductive-aged men and a prevalence as high as 20% among men who have sex with men (MSM), indicating that males cannot be ignored as a key risk group for the spread of STIs in Rwanda (Negussie et al., 2025; Rwema et al., 2022). Given the relatively low levels of comprehensive HIV knowledge among Rwandans (< 60%) (Kawuki et al., 2023; NISR et al., 2021), it is important to gain insights into male RSB in Rwanda to inform STI prevention programs and sexual health services.

There have been few studies conducted on RSB in Rwandan men; however, these studies had limitations. The study conducted by Rugigana et al. (2015) only explored HIV knowledge and its effect on risky sexual behavior among men and analyzed only two RSBs using data that are over 17 years old. A more recent qualitative study that explored social contexts as a mediator of risk behaviors was limited to Rwandan MSM (Adedimeji et al., 2019), while a 2022 study of Rwandan men was limited to MSM and transgender women (Rwema et al., 2022). Recent and more comprehensive RSB studies are needed to guide and inform policymakers as the country works towards achieving the United Nations Sustainable Development Goals. Reproductive health interventions require accurate knowledge of the patterns of sexual risk behaviors and the appropriate target groups for public health messages. This study aims to assess the prevalence of the various RSB, patterns of RSB in the population, and the factors associated with these patterns among sexually active men in Rwanda, using a recent, nationally representative study sample.

## Method

### Participants and Procedure

The Rwanda Demographic and Health Surveys (RDHS) are periodically conducted cross-sectional surveys that obtain information on demographic, health, and nutritional indicators of non-elderly adults and children. The latest survey was conducted between November 2019 and July 2020 (NISR et al., 2021).

This national survey used a stratified two-stage cluster sampling design to obtain a representative sample of 13,005 households, and details of the sampling method and data collection process are published elsewhere (NISR et al., 2021). Eligible men for the interviews were those aged 15–59 years and permanent residents of the selected households or visitors who stayed in the household the night before the survey. During the survey, 50% of men in the interviewed households were selected to respond to the men’s questionnaire (6513 males interviewed). However, given the study objectives, this secondary analysis included only men who were sexually active in the last 12 months before the survey (n = 4735). Written informed consent was provided by all respondents, and permission to access the entire RDHS database was obtained through the program website (NISR et al., 2021).

## Measures

### Independent Variables

The survey obtained various sociodemographic information using interviewer-administered questionnaires. The following is relevant to this analysis; age (15–24, 25–34, 35–44, 45 and above years), working status (yes, no), educational level (no education, primary, secondary, tertiary), marital status (married, unmarried), religion (Catholic, Protestant which included Adventist and Jehovah witness, Others which included Muslim, traditional beliefs, and others), health insurance (yes, no), mobile phone ownership (yes, no), and internet usage (yes, no). Moreover, some household information was obtained, including household size (less than six, six and above), sex of household head (male, female), place of residence (rural and urban), region of residence (Kigali, South, West, East, North), and wealth index (comprising five quintiles ranging from the poorest to the richest quintile). Additional information on recent sexual activity, being circumcised (yes, no), comprehensive knowledge of HIV (yes, no), and HIV test history (yes, no) was also obtained (Table 1). Mass media exposure was measured as a respondent having access to any of the following: radio, newspapers, and television (yes/no). Comprehensive knowledge of HIV was assessed using six items: three on HIV prevention information and three questions on misconceptions of HIV transmission modes.

**Table 1:** Background of sexually active men from the 2020 Rwanda demographic health survey (N = 4735)

Characteristics	Total sample % (n)
*Age* (years)	
15–24	17.8% (844)
25–34	30.2% (1432)
35–44	28.4% (1347)
45 and above	23.5% (1112)
*Education level*	
Tertiary or above	6.5% (310)
Secondary	17.4% (826)
Primary	64.5% (3054)
No education	11.5% (545)
*Marital status*	
Married	72.7% (3440)
Unmarried	27.3% (1295)
*Religion*	
Catholic	44.1% (2086)
Protestant (including Adventist and Jehovah’s Witness)	51.1% (2418)
Other religions (Muslim, Traditional beliefs, others)	4.9% (231)
*Working status*	
Working	95.9% (4542)
Not working	4.1% (193)
*Health insurance*	
Yes	83.1% (3937)
No	16.9% (798)
*Region of residence*	
Kigali	13.4% (633)
South	24.2% (1144)
West	22.8% (1080)
North	15.8% (749)
East	23.8% (1129)
*Household characteristics*	
Household size 6 and above	36.2% (1715)
Male head of household	89.7% (4245)
Household in urban area	23.9% (1130)
*Communications/media access*	
Mobile phone ownership	68.1% (3224)
Uses internet	21.9% (1039)
Mass media (TV, radio, newspaper) exposure	93.9% (4447)
*Human immunodeficiency virus (HIV) risk/protective factors*	
Circumcised	45.3% (2144)
Comprehensive HIV knowledge^a^	66.1% (3131)
HIV test history	71.5% (3387)

^a^3 missing values

### Dependent Variables

Four variables were used to assess sexually active men’s engagement in RSB. These were: (1) Non-condom use during sex with the most recent partner, (2) having multiple sexual partners (having more than one sex partner) in the last 12 months, (3) early sexual debut (first sexual encounter below 18 years), and (4) commercial sex engagement (defined as ever having paid for sex or having given gifts or other goods to have sex). We then explored all possible mutually exclusive combinations of the four RSBs (see Table 2), which we classified into three main themes: (1) Risky non-condom use RSB, which was when a man did not use a condom with the most recent partner and engaged in at least of one the other three RSB (multiple sex partnerships, commercial sex, and early sexual debuts), (2) RSB with condom use, engaging in at least one of the other three RSB but with condom use, and (3) Non-risky or very low RSB, which included non-condom use only (among married men who still desired to have children) and no RSB reported at all. These combinations were stratified according to regions to explore geographic patterns of RSB in Rwanda (Table 2).

**Table 2:** Distribution of risky sexual behaviors in sexually-active Rwandan men by region in 2020, based on the Demographic Health Survey

Self-reported sexual risk behaviors	All Rwanda (n = 4004) %	Kigali (n = 533) %	South (n = 938) %	West (n = 917) %	North (n = 645) %	East (n = 971) %
*Risky sexual behaviors with non-condom use*						
Non-condom use + Multiple partners + Commercial sex + Early sexual debut	0.6%	1.1%	0.3%	1.3%	0.5%	0.1%
Non-condom use + Multiple partners + Commercial sex only	0.9%	1.1%	0.3%	0.9%	1.2%	1.1%
Non-condom use + Multiple partners + Early sexual debut only	1.1%	1.7%	1.0%	1.5%	0.5%	1.0%
Non-condom use + Multiple partners only	3.3%	2.8%	3.1%	3.5%	1.9%	4.6%
Non-condom use + Commercial sex + Early sexual debut only	0.7%	0.6%	0.2%	0.9%	1.7%	0.6%
Non-condom use + Commercial sex only	2.8%	2.4%	2.5%	4.4%	1.7%	2.8%
Non-condom use + Early sexual debut only	9.6%	8.4%	9.0%	11.1%	9.9%	9.1%
Non-condom use only (among married men)^a^	72.5%	70.9%	76.5%	70.2%	72.3%	71.5%
Non-condom use only (among single men)	7.6%	10.6%	9.7%	2.9%	6.9%	7.5%
Prevalence of RSB with non-condom use among married men^a,b^	62.0%	57.8%	62.1%	66.3%	59.5%	63.3%
Prevalence of RSB with non-condom use among unmarried men	13.6%	12.8%	14.2%	19.0%	11.1%	10.7%
**Total prevalence of RSB with non-condom use % (95% CI)**^c^	**55.3%** **(53.8, 56.8)**	**45.2%** **(41.0, 49.5)**	**56.0%** **(52.8, 59.1)**	**60.3%** **(57.1, 63.4)**	**54.1%** **(50.3, 57.9)**	**56.2%** **(53.1, 59.3)**
*Risky sexual behaviors with condom use*						
Multiple partners + Commercial sex + Early sexual debut only	0.3%	0.2%	0.1%	0.5%	0.2%	0.4%
Multiple partners + Commercial sex only	0.5%	1.7%	0.4%	0.4%	0.0%	0.5%
Multiple partners + Early sexual debut only	0.7%	1.5%	0.1%	0.4%	0.6%	1.0%
Commercial sex + Early sexual debut only	0.7%	0.8%	0.5%	1.5%	0.2%	0.5%
Multiple partners only	0.8%	1.7%	0.4%	0.8%	0.6%	0.7%
Commercial sex only	0.6%	1.5%	0.2%	0.9%	0.2%	0.6%
Early sexual debut only	3.6%	5.6%	2.6%	3.6%	3.7%	3.4%
Prevalence of RSB with condom use among married men^b^	1.7%	3.6%	1.1%	1.7%	1.4%	1.4%
Prevalence of RSB with condom use among unmarried men	39.6%	36.9%	26.7%	52.6%	37.5%	44.3%
**Total prevalence of RSB but using condoms % (95% CI)**^c^	**7.2**% **(6.5, 8.1)**	**12.9**% **(10.3, 16.0)**	**4.4**% **(3.2, 5.8)**	**8.2**% **(6.5, 10.1)**	**5.4**% **(3.9, 7.4)**	**7.2**% **(5.7, 8.9)**
*Non-risky or very low risk sexual behaviors*						
Non-condom use only (among married men wishing kids)	29.3%	28.3%	31.3%	25.4%	32.9%	29.4%
No RSB reported at all	8.1%	13.5%	8.3%	6.1%	7.6%	7.2%
Prevalence of very low/no RSB among married men^b^	35.9%	38.5%	36.8%	32.0%	39.1%	35.2%
Prevalence of very low/no RSB among unmarried men	46.8%	50.3%	59.2%	28.4%	51.4%	45.0%
**Total prevalence of very low/no-risk sexual behaviors % (95% CI)**^c^	**37.5**% **(36.0, 39.0)**	**41.8**% **(37.7, 46.1)**	**39.7**% **(36.6, 42.8)**	**31.5**% **(28.6, 34.6)**	**40.5**% **(36.7, 44.3)**	**36.6**% **(33.6, 39.6)**

Missing data for condom use excluded 731 males. ^a^Excluded married men who wished to have more children, ^b^Statistically significant between married and unmarried men across all regions, ^c^Statistically significant trends across regions (i.e., with χ^2^ test *p *value < 0.05)

This study also examined the proportion of males who reported having had a STI or symptoms of an STI (genital ulcers or discharge) in the last 12 months.

### Statistical Analysis

Descriptive statistics on background characteristics and RSB patterns of the study sample were calculated. We assessed the proportion of sexually active men with each of the RSBs across the five regions of the country. Risky non-condom use RSBs, RSBs with condom use, multiple sex partnerships, commercial sex, and early sexual debut were the main outcomes, whose possible determinants were explored. The distribution (row prevalence) of these main outcome variables across various predictor variables was assessed using chi-square tests. To assess the factors associated with the outcome variables (RSBs), bivariable logistic regression analyses were first conducted, after which variables with a *p* value < 0.25 were included as candidate variables in backward multivariable logistic regression models (Dunkler et al., 2014). Respective adjusted odds ratios (*AOR*), 95% confidence intervals (CI), and *p*-values were obtained and presented, with a *p *value < 0.05 considered for conclusive statistical significance in the adjusted models. We checked for multi-collinearity among all the included predictor variables using a variance inflation factor (VIF), with a cutoff of greater than 10 (Craney & Surles, 2002). None of the factors exceeded the cutoff. We used the Statistical Package for Social Sciences (version 25.0), a complex samples package. In the analysis plan, the complex samples package incorporated the following variables to account for the multistage sampling design intrinsic to the DHS dataset: individual sample weight, sample strata for sampling errors/ design, and cluster number (Croft et al., 2018; Zou et al., 2020).

## Results

### Characteristics of Participants

The background characteristics of sexually active participants are detailed in Table 1. The majority were aged 25–44 years (59%), had only primary education (65%), were married (73%), had health insurance (83%), and resided in rural areas (76%). Almost all of the males were currently working and came from households with a male head, and about two-thirds reported less than six household members. Although 94% had exposure to mass media and over two-thirds owned a mobile phone, less than one-quarter reported internet use. Moreover, about 45% were circumcised, 66% had comprehensive knowledge of HIV, and 72% had ever tested for HIV (Table 1).

### Distribution of Risky Sexual Behaviors in Sexually Active Rwandan Men by Region

Patterns of RSBs by geographic region are detailed in Tables 2 and 3. Less than 1% of the sample reported all four RSBs. However, 55.3% of sexually active men in Rwanda did not use condoms while engaging in other RSBs or reporting not wanting children. In contrast, only 7% of sexually active Rwandan men engaged in RSB while using condoms, with men in Kigali reporting the highest prevalence (12.9%). After non-condom use (55.3%), early sexual debut was the most common RSB (17.4%) among sexually-active Rwandan males, followed by having multiple sex partners (8.3%) and then having commercial sex (7.3%).

**Table 3:** Distribution of risky sexual behaviors categories in sexually-active Rwandan men by region in 2020, based on the Demographic Health Survey

Self-reported sexual risk behaviors	All Rwanda (n = 4004) %	Kigali (n = 533) %	South (n = 938) %	West (n = 917) %	North (n = 645) %	East (n = 971) %
**Prevalence for risky non-condom use**^a^** % (95% CI)**^c^	**55.3%** **(53.8, 56.8)**	**45.2%** **(41.0, 49.5)**	**56.0%** **(52.8, 59.1)**	**60.3%** **(57.1, 63.4)**	**54.1%** **(50.3, 57.9)**	**56.2%** **(53.1, 59.3)**
Prevalence for risky non-condom use among married men^a, b^	62.5%	57.8%	62.1%	66.3%	59.5%	63.3%
Prevalence for risky non-condom use among unmarried men	13.6%	12.8%	14.2%	19.0%	11.1%	10.7%
**STI prevalence among risky non-condom use men % (95% CI)**^c^	**7.2%** **(6.1, 8.3)**	**8.3%** **(4.8, 11.8)**	**5.1%** **(3.2, 7.0)**	**6.5%** **(4.4, 8.6)**	**7.2%** **(4.4, 9.9)**	**9.5%** **(7.1, 12.0)**
*Prevalence for multiple sex partners % (95% CI)*^c^	**8.3%** **(7.4, 9.1)**	**11.8%** **(9.3, 14.7)**	**5.8%** **(4.4, 7.4)**	**9.4%** **(7.6, 11.4)**	**5.4%** **(3.9, 7.4)**	**9.6%** **(7.8, 11.5)**
Prevalence for multiple sex partners among married men^b^	6.7%	8.1%	5.0%	8.0%	4.5%	8.0%
Prevalence for multiple sex partner among unmarried men	17.5%	21.5%	10.8%	19.0%	12.5%	19.8%
**STI prevalence among those with multiple sex partners % (95% CI)**^c^	**18.7%** **(14.5, 23.0)**	**22.2%** **(11.7, 32.8)**	**24.1%** **(12.3, 35.9)**	**12.8%** **(5.6, 20.0)**	**14.3%** **(2.1, 26.5)**	**20.4%** **(12.1, 28.8)**
*Prevalence for commercial sex % (95% CI)*^c^	**7.3%** **(6.5, 8.3)**	**9.4%** **(7.1, 12.1)**	**4.6%** **(3.4, 6.0)**	**10.8%** **(8.9, 12.9)**	**5.6%** **(4.0, 7.5)**	**6.7%** **(5.2, 8.4)**
Prevalence for commercial sex among married men^b^	5.9%	6.8%	3.9%	8.5%	5.2%	5.4%
Prevalence for commercial sex among unmarried men	15.6%	16.1%	9.2%	26.7%	8.3%	15.3%
**STI prevalence among those engaging in commercial sex % (95% CI)**^c^	**16.4%** **(12.1, 20.6)**	**20.0%** **(8.5, 31.5)**	**20.9%** **(8.3, 33.6)**	**13.1%** **(6.4, 19.9)**	**13.9%** **(2.0, 25.8)**	**16.9%** **(7.6, 26.3)**
*Prevalence for early sexual debut % (95% CI)*^c^	**17.4%** **(16.2, 18.6)**	**19.9%** **(16.6, 23.4)**	**13.8%** **(11.6, 16.1)**	**20.9%** **(18.4, 23.7)**	**17.2%** **(14.4, 20.2)**	**16.2%** **(13.9, 18.6)**
Prevalence for early sexual debut among married men^b^	13.5%	14.8%	11.1%	15.7%	14.1%	12.5%
Prevalence for early sexual debut among unmarried men	40.0%	32.9%	31.7%	56.9%	41.7%	39.7%
**STI prevalence among those reporting early sexual debut % (95% CI)**^c^	**8.8%** **(6.7, 10.9)**	**11.3%** **(5.2, 17.5)**	**7.8%** **(3.1, 12.4)**	**5.2%** **(2.0, 8.4)**	**9.0%** **(3.6, 14.4)**	**12.1%** **(6.9, 17.3)**

Missing data for condom use excluded 731 males. ^a^Risky non-condom use included men who engaged in any RSB without condoms as well single men and married men who did not wish to have more children and are thereby at risk of unintended pregnancies), ^b^Statistically significant between married and unmarried men across all regions (χ^2^ test *p *value < 0.05), ^c^Statistically significant trends across regions (χ^2^ test *p *value < 0.05)

There were strong geographical variations in each of these behaviors. Notably, the Western region reported the highest levels of early sexual debut (20.9%), commercial sex (10.8%), and risky non-condom use (60.3%). The Kigali region reported the highest prevalence of multiple sexual partnerships (11.8%) but also reported the highest prevalence of very low-risk/no-risk sexual behaviors (42%). The South and North regions reported much lower levels of multiple sex partners and commercial sex than other parts of the country.

In this study sample, 6.5% of all men self-reported sexually transmitted infections (STIs) or STI symptoms (ulcers or discharge) in the past year. This prevalence was elevated slightly among males who reported non-condom use (7.2%) and among those reporting early sexual debut (8.8%) and was considerably higher among men who engaged in commercial sex (16.4%) and men with multiple sex partners (18.7%). There were significant geographic variations in the prevalence of STIs/STI symptoms among males reporting the various RSB. Notably, the prevalence was high in Kigali region, East region and South region, while the West region consistently showed lower levels of STI prevalence (Table 3). While 10.0% of males who had engaged in RSB with condom use reported an STI/STI symptoms, those who engaged in RSB with non-condom use reported a much higher prevalence (16.4%) (untabulated).

### Factors Associated with Risky Sexual Behavior with Non-Condom Use and Risky Sexual Behavior Using Condoms

The unadjusted and multivariable analyses for factors associated with RSB patterns are detailed in Tables 4 and 5. Males who engaged in RSB without condoms showed very different attributes from those who engaged in RSB with condoms. There was a significantly higher likelihood of RSB without condom use among men who were older, uncircumcised, married, with no internet access, from larger households, households headed by women, and residing in the Western region (*AOR* = 1.28–6.20, *p* < 0.05), Table 4. Among condom users, married men (*AOR* = 0.03, *p* < 0.05) and those from the Southern region (*AOR* = 0.52, *p* < 0.05) were significantly much less like to engage in RSB than unmarried men and those from Kigali, respectively, while those with secondary education were significantly more like to engage in RSB compared to those with no education (*AOR* = 2.29, *p* < 0.05), Table 5.

**Table 4:** Factors associated with risky sexual behaviors among non-condom users^c^

Factors	Row % (n)	Unadjusted odds ratio (95% CI)	*p *value^a^	Multivariable odds ratio (95% CI)	*p *value^b^
*Age* (years)					
15–24	18.2% (79)	1.00	< 0.001	1.00	** < 0.001**
25–34	36.1% (443)	2.66 (1.99, 3.54)		1.36 (0.96, 1.92)	
35–44	65.2% (838)	8.74 (6.57, 11.6)		**2.99 (2.09, 4.26)**	
45 and above	80.8% (854)	18.05 (13.3, 24.6)		**5.24 (3.57, 7.69)**	
*Education level*					
Tertiary	43.3% (103)	1.00	< 0.001	1.00	0.782
Secondary	37.4% (227)	0.80 (0.57, 1.11)		1.01 (0.67, 1.52)	
Primary	57.8% (1539)	1.88 (1.39, 2.53)		1.03 (0.66, 1.60)	
No Education	69.7% (345)	3.24 (2.28, 4.61)		1.17 (0.71, 1.90)	
*Working status*					
Not working	35.0% (36)	1.00	< 0.001	1.00	0.280
Working	55.8% (2178)	2.54 (1.62, 3.98)		1.406 (0.76, 2.61)	
*Marital status*					
Unmarried	13.6% (80)	1.00	< 0.001	1.00	** < 0.001**
Married	62.5% (2134)	10.8 (8.18, 14.1)		**6.20 (4.36, 8.81)**	
*Religion*					
Catholic	56.1% (977)	1.00	0.779	–	
Protestant	54.8% (1132)	0.95 (0.84, 1.08)			
Other religions	53.6% (105)	0.98 (0.72, 1.33)			
*Health insurance*					
Yes	55.1% (1847)	1.00	0.707	–	
No	56.4% (367)	1.03 (0.88, 1.21)			
*Wealth index quintile*					
Richest quintile	49.1% (440)	1.00	0.002	1.00	0.952
2nd wealth quintile	54.0% (457)	1.19 (0.95, 1.48)		0.93 (0.68, 1.27)	
Middle wealth quintile	59.0% (480)	1.46 (1.19, 1.80)		1.02 (0.73, 1.44)	
4th wealth quintile	58.7% (432)	1.45 (1.16, 1.80)		1.00 (0.70, 1.44)	
Poorest quintile	56.9% (405)	1.35 (1.08, 1.69)		1.01 (0.69, 1.49)	
*Residence*					
Urban	48.1% (442)	1.00	< 0.001	1.00	0.390
Rural	57.4% (1772)	1.49 (1.26, 1.77)		0.89 (0.69, 1.16)	
*Region*					
Kigali	45.2% (241)	1.00	< 0.001	1.00	**0.072**
South	56.0% (525)	1.60 (1.25, 2.04)		1.08 (0.80, 1.47)	
West	60.3% (553)	1.95 (1.53, 2.47)		**1.46 (1.07, 1.99)**	
North	54.1% (349)	1.52 (1.18, 1.96)		1.16 (0.85, 1.57)	
East	56.2% (546)	1.59 (1.26, 2.02)		1.17 (0.86, 1.58)	
*Sex of household head*					
Male	56.6% (2119)	1.00	< 0.001	1.00	**0.050**
Female	36.1% (95)	0.44 (0.32, 0.59)		**1.55 (1.00, 2.40)**	
*Household size*					
Below 6	45.6% (1156)	1.00	< 0.001	1.00	** < 0.001**
6 and above	72.1% (1058)	3.07 (2.63, 3.58)		**2.65 (2.19, 3.20)**	
*Exposure to mass media*					
Yes	55.0% (2057)	1.00	0.113	1.00	0.947
No	59.7% (157)	1.25 (0.95–1.64)		0.99 (0.72, 1.36)	
*Mobile phone ownership*					
Yes	53.3% (1481)	1.00	< 0.001	1.00	0.075
No	59.9% (733)	1.33 (1.16, 1.53)		1.17 (0.98, 1.39)	
*Internet use*					
Yes	38.1% (307)	1.00	< 0.001	1.00	**0.031**
No	59.6% (1907)	2.55 (2.17, 3.00)		**1.28 (1.02, 1.59)**	
*Circumcised*					
Yes	42.5% (712)	1.00	< 0.001	**1.00**	**0.001**
No	64.5% (1502)	2.46 (2.14, 2.84)		**1.36 (1.14, 1.63)**	
*HIV knowledge*					
High HIV knowledge	55.8% (1496)	1.00	0.422	–	
Medium/Low knowledge	54.2% (717)	0.94 (0.82, 1.09)			
*HIV test history*					
Yes	54.8% (1574)	1.00	0.186	1.00	0.132
No	56.5% (640)	1.11 (0.95, 1.29)		0.87 (0.73, 1.04)	

^a^*p* < 0.25 was used as a threshold for inclusion in multivariable analysis, ^b^*p *values for the non-significant variables in the multivariable models are shown prior to being dropped from the model, ^c^Includes respondents who reported non-condom use with any of the three other RSB (multiple sex partners, commercial sex, early sexual debut) or non-condom use among single and married men who do not want to have more children (at-risk of unintended pregnancy), HIV = Human immunodeficiency virus, Bold = significant subcategory in multivariable analysis. – Not included as a candidate variable in the backward elimination multivariable regression

**Table 5:** Factors associated with risky sexual behaviors among condom users

Factors	Row % (n)	Unadjusted odds ratio (95% CI)	*p *value^a^	Multivariable odds ratio (95% CI)	*p *value^b^
*Age* (years)					
15–24	32.8% (142)	1.00	< 0.001	1.00	0.334
25–34	7.5% (92)	0.18 (0.13, 0.24)		1.14 (0.59, 2.20)	
35–44	2.8% (36)	0.06 (0.04, 0.09)		1.54 (0.82, 2.87)	
45 and above	1.9% (20)	0.04 (0.03, 0.07)		1.77 (0.89, 3.53)	
*Education level*					
Tertiary	8.4% (20)	1.00	< 0.001	1.00	**0.009**
Secondary	17.0% (103)	2.50 (1.44, 4.33)		**2.29 (1.29, 4.08)**	
Primary	5.9% (157)	0.71 (0.43, 1.18)		1.68 (0.97, 2.89)	
No Education	2.0% (10)	0.21 (0.09, 0.48)		0.91 (0.37, 2.22)	
*Working status*					
Working	6.7% (261)	1.00	< 0.001	1.00	0.177
Not working	28.2% (29)	5.64 (3.20, 9.95)		1.61 (0.81, 3.21)	
*Marital status*					
Unmarried	39.6% (233)	1.00	< 0.001	1.00	** < 0.001**
Married	1.7% (57)	0.03 (0.02, 0.04)		**0.03 (0.02, 0.04)**	
*Religion*					
Catholic	7.5% (131)	1.00	0.044	1.00	0.484
Protestant	6.5% (134)	0.81 (0.61, 1.08)		0.84 (0.60, 1.18)	
Other religions	12.8% (25)	1.58 (0.96, 2.61)		1.12 (0.65, 1.95)	
*Health insurance*					
Yes	7.3% (245)	1.00	0.832	–	
No	6.9% (45)	0.96 (0.67, 1.38)			
*Wealth index quintile*					
Richest quintile	11.5% (103)	1.00	< 0.001	1.00	0.889
2nd wealth quintile	8.6% (73)	0.74 (0.50, 1.09)		1.26 (0.73, 2.17)	
Middle wealth quintile	5.2% (42)	0.48 (0.31, 0.73)		1.14 (0.61, 2.14)	
4th wealth quintile	5.0% (37)	0.39 (0.25, 0.61)		0.98 (0.50, 1.92)	
Poorest quintile	4.9% (35)	0.42 (0.26, 0.66)		1.08 (0.56, 2.09)	
*Residence*					
Urban	12.8% (118)	1.00	< 0.001	1.00	0.277
Rural	5.6% (172)	0.43 (0.31, 0.58)		0.78 (0.51, 1.22)	
*Region*					
Kigali	12.9% (69)	1.00	< 0.001	1.00	** < 0.001**
South	4.4% (41)	0.33 (0.22, 0.49)		**0.52 (0.31, 0.86)**	
West	8.2% (75)	0.63 (0.42, 0.94)		1.35 (0.80, 2.29)	
North	5.4% (35)	0.40 (0.25, 0.63)		0.79 (0.44, 1.41)	
East	7.2% (70)	0.62 (0.40, 0.95)		1.12 (0.64, 1.96)	
*Sex of household head*					
Male	6.0% (226)	1.00	< 0.001	1.00	0.747
Female	24.3% (64)	5.10 (3.56, 7.31)		0.93 (0.60, 1.44)	
*Household size*					
Below 6	6.9% (174)	1.00	0.091	1.00	0.234
6 and above	7.9% (116)	1.27 (0.96, 1.67)		1.22 (0.88, 1.70)	
*Exposure to mass media*					
Yes	7.5% (279)	1.00	0.251	–	
No	4.2% (11)	0.65 (0.31, 1.35)			
*Mobile phone ownership*					
Yes	7.6% (212)	1.00	0.112	1.00	0.347
No	6.4% (78)	0.78 (0.57, 1.06)		1.19 (0.83, 1.72)	
*Internet use*					
Yes	15.9% (128)	1.00	< 0.001	1.00	0.515
No	5.1% (162)	0.27 (0.21, 0.36)		0.86 (0.55, 1.35)	
*Circumcised*					
Yes	12.1% (202)	1.00	< 0.001	1.00	0.699
No	3.8% (88)	0.29 (0.21, 0.41)		0.92 (0.60, 1.41)	
*HIV knowledge*					
High HIV knowledge	7.2% (193)	1.00	0.907	–	
Medium/Low knowledge	7.3% (97)	1.02 (0.79, 1.31)			
*HIV test history*					
No	5.6% (64)	1.00	0.005	1.00	0.802
Yes	7.9% (226)	1.58 (1.15, 2.18)		1.05 (0.72, 1.52)	

^a^*p* < 0.25 was used as a threshold for inclusion in multivariable analysis, ^b^*p *values for the non-significant variables in the multivariable models are shown prior to being dropped from the model, HIV = Human immunodeficiency virus, Bold = significant subcategory in multivariable analysis. – Not included as a candidate variable in the backward elimination multivariable regression

### Factors Associated with an Early Sexual Debut, Multiple Sex Partners, and Engagement in Commercial Sex

Due to the high prevalence of early sexual debut among Rwandan men (ranging from 13.8–20.9% across regions), the associated factors of this RSB behavior were also explored in Table 6. Although a wide range of factors were associated with early sexual debut in the unadjusted analysis, the multivariable analyses revealed that early sexual debut was independently associated with: being under 25 years of age, unmarried, Protestant/Other religion, having no education, and coming from a large household (*AOR* = 1.24–2.72, *p* < 0.05).

**Table 6:** Prevalence and factors associated with early sexual debut

Factors	Row % (n)	Unadjusted odds ratio (95% CI)	*p *value^a^	Multivariable odds ratio (95% CI)	*p *value^b^
*Age* (years)					
45 and above	13.2% (139)	1.00	< 0.001	1.00	**0.001**
35–44	13.0% (167)	1.04 (0.80, 1.34)		1.03 (0.79, 1.34)	
25–34	16.8% (206)	1.35 (1.05, 1.74)		1.21 (0.92, 1.58)	
15–24	42.3% (183)	4.53 (3.35, 6.12)		**2.30 (1.62, 3.26)**	
*Education level*					
Tertiary	15.5% (37)	1.00	< 0.001	1.00	**0.097**
Secondary	25.4% (154)	2.03 (1.27, 3.25)		1.64 (0.98, 2.74)	
Primary	16.0% (425)	1.16 (0.75, 1.79)		1.56 (0.96, 2.52)	
No Education	16.0% (79)	1.27 (0.76, 2.12)		**2.01 (1.15, 3.52)**	
*Working status*					
Working	16.8% (656)	1.00	< 0.001	1.00	0.094
Not working	37.9% (39)	2.95 (1.74, 4.99)		1.61 (0.92, 2.83)	
*Marital status*					
Married	13.5% (460)	1.00	< 0.001	1.00	** < 0.001**
Unmarried	40.0% (235)	4.05 (3.26, 5.03)		**2.72 (2.10, 3.53)**	
*Religion*					
Catholic	15.2% (264)	1.00	< 0.001	1.00	**0.001**
Protestant	18.2% (375)	1.23 (1.01, 1.50)		**1.26 (1.02, 1.55)**	
Other religions	28.6% (56)	2.13 (1.45, 3.12)		**2.03 (1.40, 2.96)**	
*Health insurance*					
Yes	17.3% (581)	1.00	0.582	–	
No	17.5% (114)	0.93 (0.73, 1.19)			
*Wealth index quintile*					
Richest quintile	20.2% (181)	1.00	0.270	–	
2nd wealth quintile	17.3% (146)	0.81(0.61, 1.07)			
Middle wealth quintile	14.5% (118)	0.72 (0.54, 0.97)			
4th wealth quintile	17.4% (128)	0.86 (0.65, 1.15)			
Poorest quintile	17.1% (122)	0.84 (0.63, 1.13)			
*Residence*					
Urban	21.8% (200)	1.00	0.001	1.00	0.138
Rural	16.0% (495)	0.69 (0.55, 0.86)		0.81 (0.61, 1.07)	
*Region*					
South	13.8% (129)	1.00	0.002	1.00	**0.018**
Kigali	19.9% (106)	1.52 (1.15, 2.00)		1.21 (0.86, 1.67)	
West	20.9% (192)	1.09 (0.81, 1.45)		1.30 (0.94, 1.79)	
North	17.2% (111)	0.82 (0.61, 1.11)		1.04 (0.74, 1.46)	
East	16.2% (157)	0.80 (0.60, 1.07)		0.91 (0.65, 1.26)	
*Sex of household head*					
Male	16.1% (604)	1.00	< 0.001	1.00	0.355
Female	34.6% (91)	2.52 (1.87, 3.38)		1.18 (0.83, 1.67)	
*Household size*					
Below 6	16.4% (417)	1.00	0.028	1.00	**0.031**
6 and above	18.9% (278)	1.23 (1.02, 1.48)		**1.24 (1.02, 1.52)**	
*Exposure to mass media*					
Yes	17.6% (659)	1.00	0.155	1.00	0.297
No	13.7% (36)	0.76 (0.52, 1.11)		0.80 (0.53, 1.21)	
*Mobile phone ownership*					
Yes	17.1% (474)	1.00	0.597	–	
No	18.1% (221)	1.05 (0.87, 1.27)			
*Internet use*					
Yes	23.0% (185)		< 0.001	1.00	0.939
No	15.9% (510)	0.64 (0.52, 0.80)		0.99 (0.72, 1.35)	
*Circumcised*					
Yes	21.2% (355)	1.00	< 0.001	1.00	0.678
No	14.6% (340)	0.66 (0.55, 0.78)		1.05 (0.84, 1.30)	
*HIV knowledge*					
High HIV knowledge	17.2% (460)	1.00	0.993	–	
Medium/Low knowledge	17.8% (235)	1.00 (0.83, 1.20)			
*HIV test history*					
No	16.7% (189)	1.00	0.424	–	
Yes	17.6% (506)	1.08 (0.89, 1.32)			

^a^*p* < 0.25 was used as a threshold for inclusion in multivariable analysis. ^b^*p *values for the non-significant variables in the multivariable models are shown prior to being dropped from the model, HIV = Human immunodeficiency virus, Bold = significant subcategory in multivariable analysis, – Not included as a candidate variable in the backward elimination multivariable regression

Similar factors were noted to be associated with men's engagement in multiple-sex partnerships and commercial sex. For both of these RSBs, males who were older, unmarried, and professing a non-Christian ("Other") religion were significantly more likely to have reported these behaviors, and these males also were more likely to have had an HIV test. Additionally, males who reported multiple sex partners were also more likely to have high HIV knowledge and be from the Kigali area (Supplementary Table 1)**,** while those who reported engaging in commercial sex were more likely to reside in urban areas and the Western region (Supplementary Table 2).

## Discussion

This study examined the prevalence and patterns of various RSB as well as the associated factors among sexually active men in Rwanda. The RSB considered included non-condom use during the most recent sexual intercourse, multiple sexual partners, commercial sex, and early sexual debut. The study revealed that over one-half of the sexually active men in Rwanda engaged in condomless RSB. The observed prevalence of condomless RSB (55%) in sexually-active Rwandan males is relatively close to that reported in neighbouring countries, Uganda (45%) (Ali et al., 2021) and Tanzania (42.1%) (Reynolds et al., 2013), but higher than in Kenya (35%) (Ali et al., 2021; Darteh et al., 2020). Likewise, the high observed prevalence of early sexual debut in our study was also similar to that reported in Kenya (16%) and Uganda (15%), but much higher than that in Tanzania (7%) and Nigeria (5%) (Seff et al., 2021). Although less than 10% of sexually active Rwandan males reported commercial sex or multiple sex partners, the levels show an increasing trend from the 2015 RDHS (1.9%) and are much higher than the average of 27 African countries (4%) (Seidu et al., 2020).

The study results indicate that males who engage in RSB with condoms differ greatly from those who engage in condomless RSB, necessitating segmented public health approaches. Although men in Kigali had the highest prevalence of multiple sex partners, they also reported the highest RSB with condom use. Moreover, unmarried men and those with secondary education were more likely to engage in RSB with condom use. The association of secondary education (compared with non-educated males) with RSB using condoms was similar to previous studies (Dounebaine & Winskell, 2021; Seidu et al., 2020), although several other studies have reported higher RSB, including non-condom use among wealthier and more educated men (Berhan & Berhan, 2013, 2015; Darteh et al., 2020; Kongnyuy et al., 2006; Mutanda & Odimegwu, 2017). These findings may suggest that factors such as being single, educated, and living in the capital city may provide greater opportunities for sexual liaisons but also may provide more access to condoms and reproductive health services, including health education.

By contrast, males who reported condomless RSB appear to be older, more traditional (married, higher household size, uncircumcised), less socioeconomically advantaged (no internet access, female heads of households), and from the Western region. Older men who tend to be in stable relationships and married males may feel that condom use could be perceived as a lack of trust in their partners, regardless of whether they engage in other RSB outside their union, and our findings are similar to previous studies (Agha, 2012; Dounebaine & Winskell, 2021; Phiri et al., 2022; Reynolds et al., 2013). Internet access, especially among the youth, is shown to be a vital tool for accessing health information, which enables them to make informed sexual choices. The lack of internet access may therefore reflect a lack of health awareness or simply a proxy for lower socioeconomic circumstances (Sserwanja et al., 2023; Thin-Zaw et al., 2013). Although our findings are opposite to a previous study (Kibira et al., 2015), medical male circumcision comes with pre- and post-circumcision counselling and education on safer sex and RSB (Ensor et al., 2019), which the uncircumcised might miss out on if no other avenues are offered, a possible explanation for the observed pattern of higher condomless RSB among the uncircumcised. The geographic variations in RSB indicated that men in the Western region had the highest prevalence of commercial sex, early sexual debut, and condomless RSB. The Western region of Rwanda is characterized by large fishing communities along Lake Kivu. Fishing communities in Africa are known for the highly itinerant lifestyles of migrant workers, and these settings are documented hotspots for RSB and HIV infection (Burgos-Soto et al., 2020; Kwena et al., 2010, 2012), which might explain the observed pattern of high RSB in this area.

Large household size was shown to be a risk factor for early sexual debut and condomless RSB, which may reflect lower levels of parental supervision (Jose et al., 2012). Moreover, having a female household head was also associated with condomless RSB. We speculate that female household heads may have greater difficulty discussing sex education with male children. This might expose young males to misinformation and peer influence, thereby making them more likely to engage in RSB (Seff et al., 2021).

### Implications of Study Findings

The study findings have some practical implications for reducing RSB in the country. As compared with studies of female RSB in Rwanda, we noted that nonuse of condoms, having multiple sexual partners, and commercial sex are more prevalent among males than in females, while early sexual debut is significantly higher among females than in males. Moreover, significant regional and socio-demographic disparities in RSBs exist in both males and females in Rwanda (Sserwanja et al., 2023). Therefore, there is a need for segmenting sexual and reproductive health messages and intervention strategies, considering the different needs of each region/community. For example, promoting condom use through ensuring more reliable access and education would likely be more beneficial for men in the Western region, while awareness campaigns aimed at curbing multiple-sex partnerships and commercial sex would be more applicable to Kigali, where condoms are much more widely used.

To address the observed association between marriage and RSBs among non-condom users, we recommend implementing couple-based interventions that strengthen communication, support joint HIV/STI testing, and improve shared decision-making among married partners. Embedding structured couples’ counseling within routine reproductive and community health services may help partners discuss fidelity, risk perception, and protective behaviors against unwanted pregnancies and STIs more openly. Community-supported programs that foster trust, relationship satisfaction, and healthier conflict resolution may further reduce drivers of extramarital partnerships and associated sexual risks. These approaches can collectively help address the greater vulnerability observed among married men who do not use condoms.

Moreover, RSB education and awareness programs should target unmarried men of non-Christian religious affiliation, since they are more likely to have multiple sex partners and engage in commercial sex. Improving sex education programs, as well as engaging religious leaders, has been noted to be a useful strategy (Darteh et al., 2020; Mutanda & Odimegwu, 2017; Seff et al., 2021; Sserwanja et al., 2023). Promoting medical male circumcision would also be beneficial since it was associated with condom use, and consideration of household dynamics, such as household head and size, is warranted for successful RSB reduction campaigns and overall HIV control and prevention programs.

Although Rwanda has strived towards improving its sexual and reproductive health services, more needs to be done in addressing the observed patterns of RSB among men, especially early sexual debut, since it is not only a risk factor itself but also associated with other serious risky behaviors in adolescence throughout adulthood (Magnusson et al., 2019). Moreover, more studies are needed to fully understand the reasons behind the early sexual debut and the increasing prevalence of commercial sex among men in Rwanda.

### Strength and Limitations

We used a nationally representative study sample that obtained information across a wide range of variables, which allows a country-wide assessment of RSB in Rwandan males of all ages and a wide spectrum of socioeconomic backgrounds. However, the study has some limitations; there is a risk of social desirability since outcome responses were self-reported, and this could have affected the true estimation of RSB. There was also a lack of detailed information about sexual partnerships, such as homosexual relationships. Also, the RDHS surveys do not inquire about alcohol use during or before sex, and this RSB was therefore not included in this analysis. The levels of RSB estimated by this study may therefore be underestimated compared with other countries that include this question. Despite the limitations, the study provides useful insight into the various patterns of RSBs and the associated factors among sexually active men in Rwanda.

### Conclusions

The study found that over half of the sexually active men in Rwanda engaged in RSB without using condoms, and the patterns of RSB varied with regions and several other sociodemographic factors, which may reflect changing cultural patterns, geographic variations, and differences in access to health capital. The study results thereby highlight a need for segmenting sexual and reproductive health messages and intervention strategies, considering the different needs of each community. Segmentation of safe sex interventions should consider the geographic region, household characteristics, and demographic characteristics of the populations targeted for the various types of RSB.

## Supplementary Information

Below is the link to the electronic supplementary material.

Supplementary file1 (DOCX 20 kb)

## Data Availability

All the data supporting the findings presented in this manuscript are included in the manuscript and as supplementary information.
