# Supplementary material for: Profiles of Risky Sexual Behaviors and Associated Factors Among Sexually Active Men in Rwanda: A Nationwide Survey
Source: Arch Sex Behav. 2026 May 29;55(4):1535–48. doi: 10.1007/s10508-026-03458-6 (PMC13275783; doi:10.1007/s10508-026-03458-6)
Supplement: Supplementary file 1 — Supplementary file1 (DOCX 20 kb) [file 10508_2026_3458_MOESM1_ESM.docx]

**Supplementary Table 1** Prevalence and factors associated with having multiple sex partners

| Factors | Row % (n) | Unadjusted  odds ratio (95% CI) | *p-*value ^a^ | Multivariable  odds ratio (95% CI) | *p*-value ^b^ |
| --- | --- | --- | --- | --- | --- |
| **Age (years)** |  |  |  |  |  |
| 45 and above | 4.6% (49) | 1.00 | < 0.001 | 1.00 | **0.001** |
| 35-44 | 8.9% (115) | 2.06 (1.41, 2.99) |  | **2.02 (1.38, 2.95)** |  |
| 25-34 | 9.9% (121) | 2.31 (1.59, 3.36) |  | **1.76 (1.18, 2.62)** |  |
| 15-24 | 10.6% (46) | 2.67 (1.73, 4.10) |  | 1.07 (0.64, 1.80) |  |
| **Education level** |  |  |  |  |  |
| No Education | 6.1% (30) | 1.00 | 0.001 | 1.00 | 0.555 |
| Primary | 7.6% (202) | 1.30 (0.87, 1.93) |  | 1.03 (0.67, 1.56) |  |
| Secondary | 12.7% (77) | 2.32 (1.47, 3.65) |  | 1.32 (0.75, 2.32) |  |
| Tertiary | 9.2% (22) | 1.61 (0.80, 3.22) |  | 1.01 (0.42, 2.44) |  |
| **Working status** |  |  |  |  |  |
| Working | 8.3% (322) | 1.00 | 0.710 | -- |  |
| Not working | 8.7% (9) | 1.15 (0.55, 2.39) |  |  |  |
| **Marital status** |  |  |  |  |  |
| Married | 6.7% (229) | 1.00 | < 0.001 | 1.00 | **< 0.001** |
| Unmarried | 17.3% (102) | 3.02 (2.30, 3.96) |  | **3.27 (2.34, 4.58)** |  |
| **Religion** |  |  |  |  |  |
| Catholic | 6.9% (121) | 1.00 | 0.006 | 1.00 | **0.070** |
| Protestant | 8.8% (182) | 1.24 (0.94, 1.63) |  | 1.16 (0.87, 1.53) |  |
| Other religions | 14.3% (28) | 2.08 (1.32, 3.28) |  | **1.77 (1.08, 2.88)** |  |
| **Health insurance** |  |  |  |  |  |
| Yes | 8.1% (272) | 1.00 | 0.688 | -- |  |
| No | 9.1% (59) | 1.06 (0.79, 1.42) |  |  |  |
| **Wealth index quintile** |  |  |  |  |  |
| Poorest quintile | 7.2% (51) | 1.00 | 0.010 | 1.00 | 0.407 |
| 4^th^ wealth quintile | 5.8% (43) | 0.73 (0.48, 1.11) |  | 0.72 (0.46, 1.13) |  |
| Middle wealth quintile | 7.9% (64) | 1.15 (0.78, 1.69) |  | 1.05 (0.67, 1.65) |  |
| 2^nd^ wealth quintile | 8.6% (73) | 1.29 (0.86, 1.94) |  | 1.01 (0.61, 1.66) |  |
| Richest quintile | 11.2% (100) | 1.48 (1.00, 2.20) |  | 0.99 (0.56, 1.74) |  |
| **Residence** |  |  |  |  |  |
| Rural | 7.5% (231) | 1.00 | < 0.001 | 1.00 | 0.139 |
| Urban | 10.9% (100) | 1.63 (1.25, 2.12) |  | 1.27 (0.92, 1.76) |  |
| **Region** |  |  |  |  |  |
| Kigali | 11.8% (63) | 1.00 | < 0.001 | 1.00 | **0.002** |
| South | 5.8% (54) | 0.52 (0.35, 0.78) |  | 0.68 (0.45, 1.04) |  |
| West | 9.4% (86) | 0.91 (0.65, 1.29) |  | 1.25 (0.87, 1.79) |  |
| North | 5.4% (35) | 0.43 (0.26, 0.71) |  | **0.59 (0.36, 0.97)** |  |
| East | 9.6% (93) | 0.90 (0.63, 1.29) |  | 1.20 (0.82, 1.75) |  |
| **Sex of household head** |  |  |  |  |  |
| Male | 7.8% (291) | 1.00 | < 0.001 | 1.00 | 0.113 |
| Female | 15.2% (40) | 2.41 (1.56, 3.71) |  | 1.51 (0.91, 2.51) |  |
| **Household size** |  |  |  |  |  |
| Below 6 | 8.5% (215) | 1.00 | 0.638 | -- |  |
| 6 and above | 7.9% (116) | 0.94 (0.71, 1.23) |  |  |  |
| **Exposure to mass media** |  |  |  |  |  |
| Yes | 8.3% (310) | 1.00 | 0.757 | -- |  |
| No | 8.0% (21) | 0.93 (0.58, 1.48) |  |  |  |
| **Mobile phone ownership** |  |  |  |  |  |
| Yes | 9.1% (254) | 1.00 | 0.002 | 1.00 | 0.085 |
| No | 6.3% (77) | 0.65 (0.49, 0.86) |  | 0.78 (0.58, 1.04) |  |
| **Internet use** |  |  |  |  |  |
| Yes | 11.8% (95) | 1.00 | 0.007 | 1.00 | 0.158 |
| No | 7.4% (236) | 0.67 (0.50, 0.90) |  | 1.28 (0.91, 1.79) |  |
| **Circumcised** |  |  |  |  |  |
| Yes | 10.4% (174) | 1.00 | 0.001 | 1.00 | 0.579 |
| No | 6.7% (157) | 0.66 (0.51, 0.84) |  | 1.09 (0.80, 1.49) |  |
| **HIV knowledge** |  |  |  |  |  |
| High HIV knowledge | 9.0% (242) | 1.00 | 0.035 | 1.00 | **0.035** |
| Medium/Low knowledge | 6.7% (89) | 0.75 (0.57, 0.98) |  | **0.75 (0.57, 0.98)** |  |
| **HIV test history** |  |  |  |  |  |
| No | 4.9% (56) | 1.00 | < 0.001 | 1.00 | **< 0.001** |
| Yes | 9.6% (275) | 1.86 (1.38, 2.50) |  | **1.76 (1.30, 2.38)** |  |
| ^a^ *p* < 0.25 was used as a threshold for inclusion in multivariable analysis, ^b^ *p*-values for the non-significant variables in the multivariable models are shown prior to being dropped from the model, HIV = Human immunodeficiency virus, Bold = significant subcategory in multivariable analysis, -- Not included as a candidate variable in the backward elimination multivariable regression | | | | | |

**Supplementary Table 2** Prevalence and factors associated with commercial sex

|  | Row % (n) | Unadjusted  odds ratio (95% CI) | *p*-value ^a^ | Multivariable  odds ratio (95% CI) | *p*-value ^b^ |
| --- | --- | --- | --- | --- | --- |
| **Age** |  |  |  |  |  |
| 45 and above | 5.5% (58) | 1.00 | 0.049 | 1.00 | **0.011** |
| 35-44 | 7.5% (97) | 1.41 (1.00, 1.98) |  | 1.37 (0.97, 1.91) |  |
| 25-34 | 8.0% (98) | 1.53 (1.09, 2.14) |  | 1.13 (0.79, 1.62) |  |
| 15-24 | 9.2% (40) | 1.59 (1.02, 2.48) |  | **0.56 (0.32, 0.98)** |  |
| **Education level** |  |  |  |  |  |
| No Education | 6.3% (31) | 1.00 | 0.380 | -- |  |
| Primary | 7.0% (186) | 1.09 (0.73, 1.64) |  |  |  |
| Secondary | 9.2% (56) | 1.31 (0.82, 2.08) |  |  |  |
| Tertiary | 8.4% (20) | 1.51 (0.87, 2.60) |  |  |  |
| **Working status** |  |  |  |  |  |
| Working | 7.3% (285) | 1.00 | 0.599 | -- |  |
| Not working | 7.8% (8) | 0.81 (0.37, 1.77) |  |  |  |
| **Marital status** |  |  |  |  |  |
| Married | 5.9% (201) | 1.00 | < 0.001 | 1.00 | **< 0.001** |
| Unmarried | 15.6% (92) | 2.71 (1.99, 3.69) |  | **3.49 (2.30, 5.30)** |  |
| **Religion** |  |  |  |  |  |
| Catholic | 6.3% (110) | 1.00 | 0.033 | 1.00 | **0.143** |
| Protestant | 7.6% (157) | 1.10 (0.83, 1.45) |  | 1.05 (0.79, 1.40) |  |
| Other religions | 13.3% (26) | 1.97 (1.19, 3.28) |  | **1.64 (1.00, 2.70)** |  |
| **Health insurance** |  |  |  |  |  |
| Yes | 7.1% (239) | 1.00 | 0.412 | -- |  |
| No | 8.3% (54) | 1.15 (0.82, 1.63) |  |  |  |
| **Wealth index quintile** |  |  |  |  |  |
| Poorest quintile | 5.9% (42) | 1.00 | 0.002 | 1.00 | 0.350 |
| 4^th^ wealth quintile | 5.2% (38) | 0.71 (0.44, 1.14) |  | 0.75 (0.46, 1.20) |  |
| Middle wealth quintile | 7.0% (57) | 1.07 (0.68, 1.67) |  | 1.13 (0.72, 1.77) |  |
| 2^nd^ wealth quintile | 7.6% (64) | 1.03 (0.64, 1.67) |  | 0.91 (0.55, 1.50) |  |
| Richest quintile | 10.3% (92) | 1.57 (1.04, 2.36) |  | 1.14 (0.68, 1.89) |  |
| **Residence** |  |  |  |  |  |
| Rural | 6.1% (187) | 1.00 | < 0.001 | 1.00 | **< 0.001** |
| Urban | 11.5% (106) | 2.15 (1.65, 2.82) |  | **2.18 (1.60, 2.96)** |  |
| **Region** |  |  |  |  |  |
| Kigali | 9.4% (50) | 1.00 | < 0.001 | 1.00 | **< 0.001** |
| South | 4.6% (43) | 0.50 (0.32, 0.78) |  | 0.99 (0.60, 1.65) |  |
| West | 10.8% (99) | 1.30 (0.88, 1.91) |  | **2.69 (1.74, 4.17)** |  |
| North | 5.6% (36) | 0.58 (0.36, 0.96) |  | 1.20 (0.67, 2.16) |  |
| East | 6.7% (65) | 0.74 (0.48, 1.14) |  | 1.54 (0.95, 2.51) |  |
| **Sex of household head** |  |  |  |  |  |
| Male | 6.9% (257) | 1.00 | < 0.001 | 1.00 | 0.152 |
| Female | 13.7% (36) | 2.27 (1.48, 3.48) |  | 1.48 (0.86, 2.54) |  |
| **Household size** |  |  |  |  |  |
| Below 6 | 7.7% (196) | 1.00 | 0.334 | -- |  |
| 6 and above | 6.6% (97) | 0.87 (0.66, 1.15) |  |  |  |
| **Exposure to mass media** |  |  |  |  |  |
| Yes | 7.4% (278) | 1.00 | 0.514 | -- |  |
| No | 5.7% (15) | 0.81 (0.43, 1.52) |  |  |  |
| **Mobile phone ownership** |  |  |  |  |  |
| Yes | 7.6% (210) | 1.00 | 0.667 | -- |  |
| No | 6.8% (83) | 0.94 (0.69, 1.26) |  |  |  |
| **Internet use** |  |  |  |  |  |
| Yes | 10.2% (82) | 1.00 | 0.012 | 1.00 | 0.428 |
| No | 6.6% (211) | 0.67 (0.50, 0.92) |  | 1.18 (0.78, 1.79) |  |
| **Circumcised** |  |  |  |  |  |
| Yes | 9.7% (162) | 1.00 | < 0.001 | 1.00 | 0.562 |
| No | 5.6% (131) | 0.60 (0.47, 0.77) |  | 0.90 (0.62, 1.29) |  |
| **HIV knowledge** |  |  |  |  |  |
| High HIV knowledge | 7.2% (192) | 1.00 | 0.465 | -- |  |
| Medium/Low knowledge | 7.6% (101) | 1.11 (0.83, 1.49) |  |  |  |
| **HIV test history** |  |  |  |  |  |
| No | 5.4% (61) | 1.00 | 0.004 | 1.00 | **0.046** |
| Yes | 8.1%(232) | 1.51 (1.14, 2.00) |  | **1.35 (1.00, 1.80)** |  |
| ^a^  *p* < 0.25 was used as a threshold for inclusion in multivariable analysis, ^b^ *p*-values for the non-significant variables in the multivariable models are shown prior to being dropped from the model, HIV = Human immunodeficiency virus, Bold = significant subcategory, -- Not included as a candidate variable in the backward elimination multivariable regression | | | | | |
